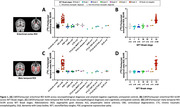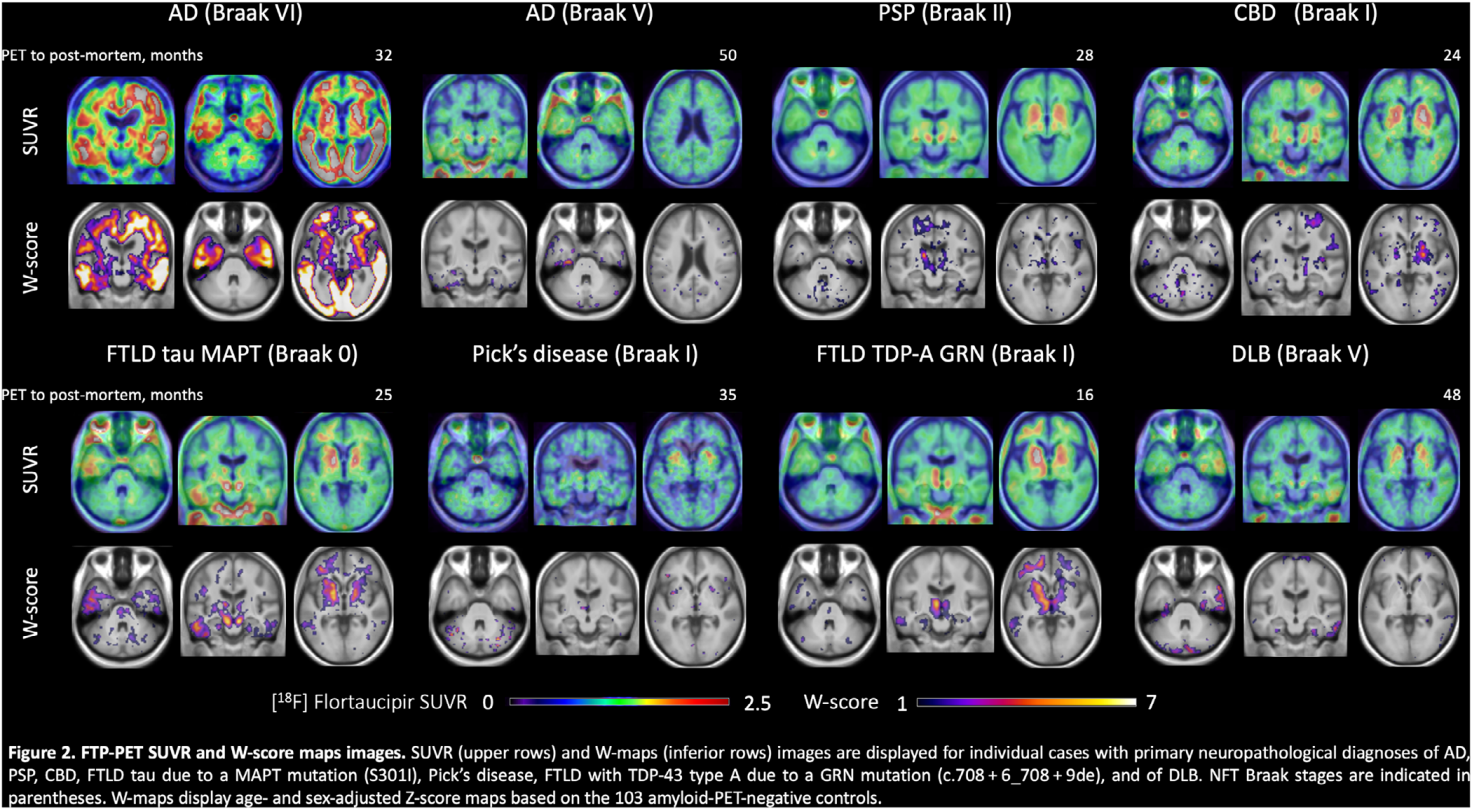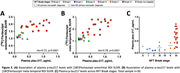# Association of ^18^F‐flortaucipir PET with tau neuropathology in AD and other neurodegenerative disorders

**DOI:** 10.1002/alz.092150

**Published:** 2025-01-09

**Authors:** Agathe Vrillon, Jhony Alejandro Mejía‐Perez, Salvatore Spina, Daniel R. Schonhaut, Claire Yballa, David N. soleimani‐meigooni, Adam L. Boxer, Jeffrey L. Dage, Julio C. Rojas, Lawren VandeVrede, Argentina Lario Lago, William J. Jagust, Bruce L. Miller, Howard J. Rosen, William W. Seeley, Lea T. Grinberg, Gil D. Rabinovici, Renaud La Joie

**Affiliations:** ^1^ Memory and Aging center, UCSF, San Francisco, CA USA; ^2^ University of California, San Francisco, San Francisco, CA USA; ^3^ Memory and Aging Center, UCSF Weill Institute for Neurosciences, University of California San Francisco, San Francisco, CA USA; ^4^ Memory and Aging Center, Weill Institute for Neurosciences, University of California, San Francisco, San Francisco, CA USA; ^5^ Memory and Aging Center, UCSF Weill Institute for Neurosciences, San Francisco, CA USA; ^6^ Indiana Alzheimer's Disease Research Center, Indianapolis, IN USA; ^7^ Memory and Aging Center, UCSF Weill Institute for Neurosciences, University of California, San Francisco, San Francisco, CA USA; ^8^ UCSF Weill Institute for Neurosciences, University of California, San Francisco, CA USA; ^9^ Memory & Aging Center, Department of Neurology, University of California in San Francisco, San Francisco, CA USA

## Abstract

**Background:**

Tau‐PET with [18F]Flortaucipir is FDA‐approved for the identification of AD tau neuropathology in the differential diagnosis of patients with cognitive impairment. However, its performance in detecting early AD stages requires further assessment. We aimed to i) examine the relationships between Flortaucipir‐PET and AD neuropathology, and ii) characterize the relationship between Flortaucipir‐PET and emerging plasma ptau217 biomarker in autopsy cases.

**Method:**

We analyzed Flortaucipir‐PET consecutively acquired from 59 patients with a clinical diagnosis of various neurodegenerative diseases who underwent post‐mortem examination at the UCSF Alzheimer’s Disease Research Center (age=66±12, 32% female, median PET‐to‐autopsy=45 months, range 25‐60). Flortaucipir‐PET was acquired 80‐100 min post‐injection and processed using an MRI‐based pipeline to create Standardized Uptake Value Ratio (SUVR, reference: inferior cerebellar cortex). Amyloid‐PET‐negative cognitively unimpaired participants (n=103) without autopsy data were included as a reference. We examined the associations between i) Flortaucipir‐SUVR in two regions of interest (ROI): the temporal meta‐ROI (i.e. validated AD signature measure) and entorhinal cortex (i.e. early tau region), and ii) neuropathological diagnosis and neurofibrillary tangle Braak stages. Plasma p‐tau217 measurement on the Meso Scale Discovery platform was available for a sample of n=31 (median PET‐to‐plasma=3.5 months, range 1‐9).

**Results:**

Our cohort included 30 patients with a primary neuropathological diagnosis of AD, 23 with various non‐AD tauopathies and 4 patients with non‐tau diseases. Flortaucipir‐SUVR was elevated in patients with primary AD neuropathology (Figure 1a,c) and differentiated them from non‐AD cases with high accuracy (areas under the curve>0.98 for both regions). When examining associations between Braak stage and Flortaucipir‐SUVR, we only detected consistent PET signal in patients with Braak VI (Figure 1b,d). Patients with both non‐AD tauopathies and non‐tau diseases displayed variable patterns of mild tracer uptake (Figure 2). Plasma p‐tau217 showed strong association with Flortaucipir‐SUVR (r≥0.73), fully driven by cases with Braak stages V‐VI (Figure 3).

**Conclusion:**

[18F]flortaucipir‐PET can identify advanced AD‐tau neuropathology and is highly concordant with plasma p‐tau217. The lack of evidence for early‐stage detection should be interpreted with caution due to the low number of cases with Braak stages III‐V, and the delay between PET and autopsy.